# High prevalence of IgE sensitization to inactivated influenza vaccines, yet robust IgG4 responses, in a healthy pediatric population

**DOI:** 10.1111/irv.13053

**Published:** 2022-09-09

**Authors:** Prince Baffour Tonto, Mizuho Nagao, Shigeru Suga, Kiyosu Taniguchi, Masahiro Hirayama, Tetsuo Nakayama, Takuji Kumagai, Takao Fujisawa, the Influenza Vaccine Study Group

**Affiliations:** ^1^ Allergy Center and Infectious Disease Center National Hospital Organization Mie National Hospital Tsu Japan; ^2^ Department of Child Health and Development Mie University Graduate School of Medicine Tsu Japan; ^3^ Department of Pediatrics Mie University Graduate School of Medicine Tsu Japan; ^4^ Omura Satoshi Memorial Institute Kitasato University Graduate School of Infection Control Sciences Tokyo Japan; ^5^ Kumagai Pediatric Clinic Hokuto Japan

**Keywords:** anaphylaxis, influenza vaccine, specific IgE, specific IgG4, specific IgG4/IgE ratio

## Abstract

**Background:**

Anaphylaxis following influenza vaccination is a rare but serious problem. The underlying immune responses are not well understood. This study elucidated the IgE and IgG antibody responses in healthy children and adolescents following inactivated influenza vaccines (IIVs).

**Methods:**

The efficacy and safety of quadrivalent IIV (QIV) and trivalent IIV (TIV) were compared in healthy subjects aged 0–18 years. Serum IIV‐specific IgE, IgG, and IgG4 levels (sIgE, sIgG, and sIgG4) were measured with ImmunoCAP. Hemagglutination inhibition (HI) assay was performed for each influenza virus subtype. Sera from earlier patients who developed anaphylaxis to different IIVs were similarly tested.

**Results:**

A total of 393 subjects were enrolled: 96 were 6 months−2 years old, 100 were 3–5 years old, 100 were 6–12 years old, and 97 were 13–18 years old. No anaphylaxis was observed. Generally, QIV and TIV induced similar antibody responses. IIV‐sIgE levels rose significantly after vaccination in the 6 months–2 years old and 3–5 years old groups, did not change in the 6–12 years old group, and decreased in the 13–18 years old group. In contrast, the IIV‐sIgG4/sIgE ratio increased significantly after vaccination in all age groups. Sensitized subjects had significantly higher HI titers and IIV‐sIgG levels in the youngest age group and higher IIV‐sIgG4 levels in all age groups compared with the non‐sensitized. The IIV‐sIgG4/sIgE ratio in five patients with anaphylaxis was significantly lower than in age‐matched healthy subjects.

**Conclusion:**

IIVs induce IgE sensitization in healthy children but also robust IgG4 responses that may protect them from anaphylaxis.

**Abbreviations**
HA: hemagglutinin
IIV: inactivated influenza vaccine
IVA: influenza vaccine‐associated anaphylaxis
QIV: quadrivalent influenza vaccine
sIgE: specific IgE
sIgG: specific IgG
sIgG4: specific IgG4
TIV: trivalent influenza vaccine
y: years

## 1 INTRODUCTION

Immunization programs are vital public health interventions to reduce the disease burden caused by infectious pathogens. Nonetheless, growing hesitation on immunization because of adverse reactions threatens to hamper future immunization programs. Inactivated influenza vaccines (IIVs) used to control and manage seasonal influenza can cause minor and severe adverse reactions, the latter serving as a contraindication to future immunization. One of the severe adverse events (AEs) that can occur following influenza vaccination is anaphylaxis, a rare and life‐threatening immediate hypersensitivity reaction.^1^ Influenza vaccines are administered to a large number of people annually, and the cumulative incidence of anaphylaxis has been the highest among all vaccines.^2^

Previously, egg allergy was thought to represent a high risk for anaphylaxis because vaccine strains are propagated in the allantoic cavity of embryonated chicken eggs and the final products contain a trace amount of egg proteins that might cause an egg‐allergic patient to develop anaphylaxis; however, cumulative evidence now clearly demonstrates the safety of IIV for individuals with egg allergy of any severity.^3^ Instead, we found that hemagglutinin (HA) protein, the active ingredient in IIV, was a causal allergen of anaphylaxis.^4^ During a surge in IIV‐associated anaphylaxis (IVA) in young children aged 3–8 years old in Japan in the 2011–2012 influenza season, we observed high IgE responses to HA in IVA patients but not in healthy or egg‐allergic patients without any AEs.^4^ This suggests that production of IgE antibodies to IIV may be a central issue.

To date, studies on the mechanism of IVA and the presence of IgE antibodies following influenza vaccination have been few and are mostly case‐series or small‐size studies.^5^, ^6^, ^7^, ^8^ In addition, immune responses to vaccination that may be related to anaphylaxis are not well understood. Therefore, there is a need for further, large‐scale studies to evaluate IgE sensitization and immune responses to influenza vaccines that might shed light on the mechanism of IVA and lead to the production of safer vaccines.

The present study was a multicenter clinical trial that evaluated the IgE and IgG antibody responses to IIVs in healthy Japanese children and adolescents.

## 2 METHODS

### 2.1 Vaccines

The vaccines used in this study were trivalent IIV (TIV) and quadrivalent IIV (QIV). The IIVs were produced by inoculating the virus into the allantoic cavity of embryonated hen eggs and subsequently inactivated with formaldehyde. They were produced at four locations: the Chemo‐Sero‐Therapeutic Research Institute (currently, KM Biologics Co., Ltd.; Kumamoto, Japan), Daiichi Sankyo Co., Ltd. (Tokyo, Japan), the Research Foundation for Microbial Diseases of Osaka University (Osaka, Japan), and Denka Co., Ltd. (Tokyo, Japan). Each 1‐ml vial of TIV contained ≥30 μg of HA protein from each of the following viral strains, which were recommended for the 2014/2015 influenza season in the Northern hemisphere: A/California/7/2009 (H1N1) pdm09‐like virus, A/New York/39/2012 (H3N2), and B/Massachusetts/2/2012‐like virus. Each 1‐ml vial of QIV contained ≥30 μg of HA protein from each of those three viral strains plus B/Brisbane/60/2008. The vaccines were administered at 0.25 ml/dose to children 6 months to 2 years of age and 0.5 ml/dose to children 3 years of age and older.

### 2.2 Clinical study design

This study was designed (1) to compare the immunogenicity and safety of TIV and QIV because of their difference in protein content (more protein in QIV) and (2) to investigate the IgE and IgG responses related to allergic sensitization to the vaccines (UMIN000015324). The study was conducted at 11 centers located throughout Japan during the 2014–2015 influenza season. The study was approved by the ethics committee of Mie National Hospital and conducted in compliance with the study protocol (approval numbers: 26‐13 and 27‐11). Guardians of all subjects provided informed consent. Healthy volunteers aged between 0 and 18 years were enrolled in the study and categorized into four age groups: 6 months–2 years, 3–5 years, 6–12 years, and 13–18 years. The subjects in each age group were randomized to receive a subcutaneous injection of either TIV or QIV at a 1:2 allocation ratio. Subjects younger than 13 years old received two doses of either TIV or QIV at a 4‐week interval, whereas those in the 13–18 years old group received a single dose of either vaccine.

### 2.3 Sample collection and antibody measurement

Blood samples were collected before the first dose and 4 weeks after each dose. Influenza vaccine‐specific IgE (sIgE), IgG (sIgG), and IgG4 (sIgG4) were measured using the ImmunoCAP® assay system (Phadia AB, Uppsala, Sweden). QIV, as an influenza vaccine antigen, was covalently coupled to the activated solid phase through the amino groups of the proteins according to standard ImmunoCAP methodology. The data were expressed as kilounits per liter (kU_A_/L) for specific IgE and milligrams per liter (mgA/L) for specific IgG and IgG4. IgE positivity was defined as an IgE titer >0.1 kUA/L, which is the lower limit of detection and is considered to have the potential to cause hypersensitivity reactions, such as anaphylaxis. The hemagglutination inhibition (HI) assay^9^ was performed to test for the presence of putative protective antibodies against the influenza virus strains contained in the vaccines.

### 2.4 Safety

Local and systemic AEs were recorded by the subjects' parents in a diary within 7 days after each vaccine dose.

### 2.5 Statistical analysis

Demographic data were compared among the age groups using the Kruskal–Wallis test (continuous data) or chi‐square test (categorical data). The Mann–Whitney test was performed to compare antibody levels between two independent groups. Wilcoxon's signed‐rank test or Friedman's test with Dunn's post‐test were performed to compare changes in antibody levels before and after vaccination. Logistic regression analysis was performed to estimate associations between IgE sensitization and background factors, including the age group, gender, history of influenza infection, previous influenza vaccination, and history of allergic diseases. Statistical analyses were carried out using Graph Pad Prism 9.3 and IBM SPSS 24.0 statistics software.

### 2.6 Role of the funding sources

The study was funded by unconditional grants from the Chemo‐Sero‐Therapeutic Research Institute (currently, KM Biologics Co., Ltd.), Daiichi Sankyo Co., Ltd., the Research Foundation for Microbial Diseases of Osaka University, and Denka Co., Ltd. The sponsors had no role in data monitoring, statistical analysis, or data interpretation. The authors were responsible for the study design, data collection, analysis, interpretation of the data, and writing of the report. The authors had complete independence over the conduct, integrity, and publication of the study.

## 3 RESULTS

### 3.1 Characteristics of the subjects

The study enrolled a total of 393 volunteers, about 100 in each age group (Table 1). The gender ratio showed slight male predominance in all age groups. The proportion of the subjects with a history of previous influenza infection was 19.8% in the 6 months–2 years group and increased in the older age groups, up to 69.1% in the 13–18 years group. Subjects who had no previous influenza vaccination were 40% in the 6 months–2 years group and 11%, 7%, and 2% in the three older age groups, respectively. The number of subjects with physician‐diagnosed allergic diseases was similar in each of the four age groups, with asthma at about 30%, allergic rhinitis at 50%, and egg allergy at less than 10% (Table 1).

**TABLE 1:** Baseline characteristics of participants

	Age group			
	6 months–2 years (*n* = 96)	3–5 years (*n* = 100)	6–12 years (*n* = 100)	13–18 years (*n* = 97)
Gender, male, *n* (%)	50 (52.1)	51 (51.0)	53 (53.0)	50 (51.6)
Previous influenza infection, *n* (%)	19 (19.8)	45 (44.6)	67 (67.0)	67 (69.1)
In last season, *n* (%)	12 (12.5)	26 (26.0)	18 (18.0)	9 (9.28)
In other seasons, *n* (%)	7 (7.3)	19 (19.0)	49 (49.0)	58 (59.8)
Type A, *n* (%)	15 (15.6)	30 (30.0)	31 (31.0)	31 (32.0)
Type B, *n* (%)	3 (3.1)	7 (7.0)	6 (6.0)	3 (3.1)
Type A and B, *n* (%)	1 (1.0)	7 (7.0)	30 (30.0)	33 (34.0)
Number of previous vaccinations, median (range)	0 (0–4)	5.5 (0–16)	13 (0–24)	16 (0–28)
Percentage with no previous vaccination, %	40%	11%	7%	2%
Physician‐diagnosed allergic diseases				
Bronchial asthma, *n* (%)	27 (28.1)	34 (34.0)	28 (28.0)	26 (26.8)
Atopic dermatitis, *n* (%)	14 (14.6)	11 (11.0)	13 (13.0)	12 (12.4)
Allergic rhinitis, *n* (%)	36 (37.5)	44 (44.0)	48 (48.0)	55 (56.7)
Food allergy, *n* (%)	11 (11.5)	9 (9.0)	8 (8.0)	11 (11.3)
Egg allergy, *n* (%)	8 (8.3)	8 (8.0)	4 (4.0)	6 (6.2)

### 3.2 Safety

Tables S1 and S2 summarize the AEs that occurred within 7 days after vaccination. Generally, no severe AEs, including anaphylaxis, were observed. There were also no AEs that were immediate allergic reactions, such as urticaria. Despite the concern that QIV would cause more AEs than TIV because of its higher protein content, the incidence of AEs did not differ between TIV and QIV (Table S1). The distribution of AEs differed among the four age groups, as is often experienced in general practice (Table S2). The results were comparable to those in a recent meta‐analysis.^10^

### 3.3 Similar immunogenicity of TIV and QIV

Next, we compared TIV and QIV in terms of the HI geometric mean titer (Table S3), geometric mean ratio (Table S4), seroconversion rate (Table S5), and seroprotection rate (Table S6)^11^ for each constituent influenza virus strain. The immunogenicities of the vaccines were similar, excluding the response to B/Victoria, which was not contained in TIV, as reported previously.^12^ Likewise, the TIV and QIV groups showed no significant differences in either the pre‐ or post‐vaccination levels of IIV‐sIgE (Figure S1). The IIV‐sIgG and IIV‐sIgG4 levels were also similar between TIV and QIV (data not shown). Therefore, in subsequent analyses, we integrated the TIV and QIV data.

### 3.4 Influenza vaccine‐specific IgE antibody

**FIGURE 1:**
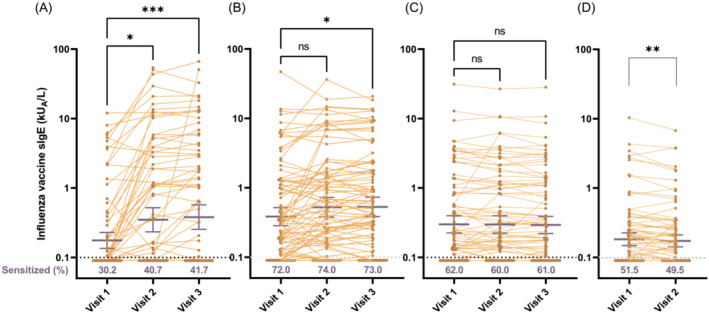
Prevalence of sensitization to influenza vaccine and changes in influenza vaccine‐specific IgE antibody following vaccination. Sensitization was defined as a specific IgE (sIgE) level to the influenza vaccine (QIV > 0.1 kU_A_/L). The percentages of sensitized subjects before (Visit 1), at 4 weeks after the first dose (Visit 2), and at the second dose (Visit 3) for four age groups: (A) 6 months to 2 years; (B) 3 to 5 years; (C) 6 to 12 years; and (D) 13 to 18 years, are shown in the bottom of each graph in red letter. The level of influenza vaccine‐specific IgE following vaccination increased significantly in age groups of (A) 6 months to 2 years and (B) 3 to 5 years, did not change in (C), 3 to 5 years, and decreased significantly in (D), 13 to 18 years old. Gridlines indicate the lower limit of detection of specific IgE. *p < 0.05, **p < 0.01, and ***p < 0.001, Wilcoxon signed‐rank test or Friedman test, followed by Dunn's multiple comparison test. IIV, inactivated influenza vaccines; QIV, quadrivalent IIV

We evaluated the IIV‐sIgE level in each age group. The percentage of sensitized children (sIgE > 0.1 kU_A_/L) increased from 30% to 43% in the 6 months–2 years group following vaccination (Figure 1A, bottom). Although the percentage of sensitization did not change after vaccination in the older age groups, more than half of the subjects were found to be sensitized, above 70% in the 3–5 years group, and about 60% and 50% in the 6–12 years and 13–18 years groups, respectively (Figure 1B–D).

We also observed a significant increase in the IIV‐sIgE level after the first and second vaccine doses in the 6 months–2 years group (Figure 1A). In the 3–5 years group, a significant increase was observed after the second dose (Figure 1B). No post‐vaccination increase was seen in the 6–12 years group (Figure 1C). Interestingly, the IIV‐sIgE level decreased significantly in the 13–18 years group (Figure 1D).

### 3.5 Factors associated with IgE sensitization to the influenza vaccines

Logistic regression analysis was used to explore the relative importance of age, gender, history of influenza infection, previous influenza vaccination, history of physician‐diagnosed asthma, atopic dermatitis, allergic rhinitis, and food allergy as risk factors for IgE sensitization to the influenza vaccines. Univariate analysis showed that subjects in the 6 months–2 years and 3–5 years age groups and those with physician‐diagnosed asthma and allergic rhinitis were likely to be sensitized. After adjusting for all the covariates, the 3–5 years group (aOR, 3.584; 95% CI, 1.701–7.549; *p* = 0.001) and allergic rhinitis (aOR, 1.749; 95% CI, 1.020–2.999; *p* = 0.042) were identified as significant risk factors (Table S7).

### 3.6 Influenza vaccine‐induced antibody responses

**FIGURE 2:**
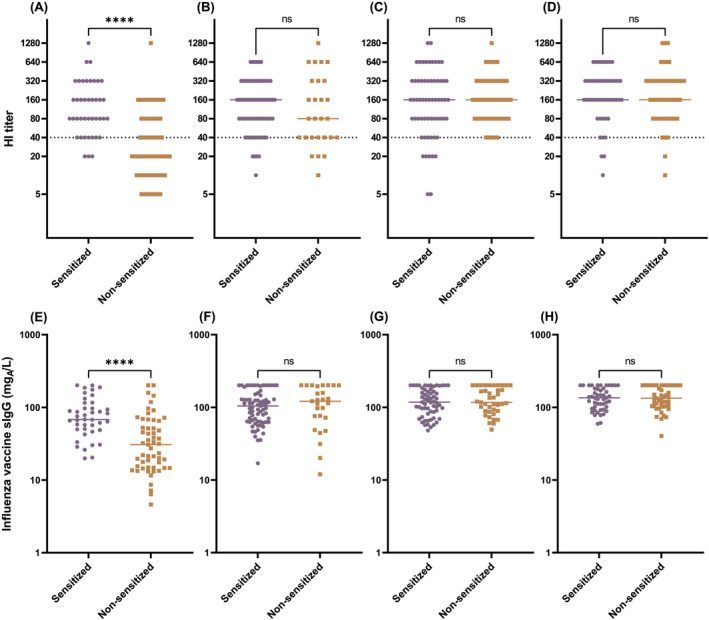
Comparison of HI titer (A–D) and influenza vaccine‐specific IgG (E–H) after vaccination between sensitized and non‐sensitized subjects in age groups of 6 months to 2 years (A, E), 3 to 5 years (B, F), 6 to 12 years (C, G), and 13 to 18 years (D, H). Gridlines indicate the putative protection level. ****p < 0.0001; ns, not significant; Mann–Whitney test

Because patients with atopic asthma were reported to show impaired immune responses to respiratory virus infections,^13^ sensitized subjects might be at a disadvantage in terms of acquiring vaccine protection against infection. But contrary to this idea, sensitized subjects in the youngest age group, a majority of whom were naïve to influenza, had a significantly higher HI titer and IIV‐sIgG level after vaccination (Figure 2A,E; Figure S2) compared with their non‐sensitized counterparts. No similar differences were seen in the older age groups (Figure 2B–D,F–H).

### 3.7 Influenza vaccine‐specific IgG4 and the sIgG4/sIgE ratio

**FIGURE 3:**
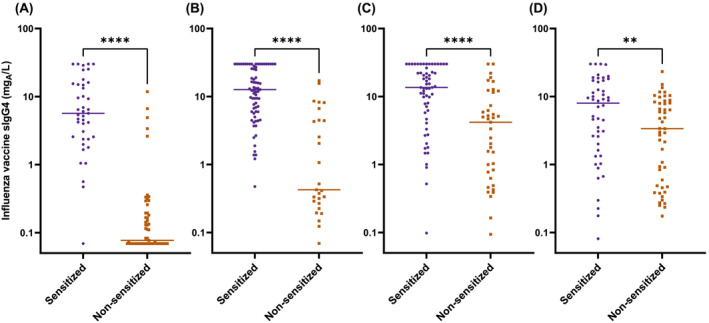
Comparison of influenza vaccine‐specific IgG4 after vaccination between sensitized and non‐sensitized subjects in age groups of 6 months to 2 years (A), 3 to 5 years (B), 6 to 12 years (C), and 13 to 18 years (D). **p < 0.01 and ****p < 0.0001; Mann–Whitney test

**FIGURE 4:**
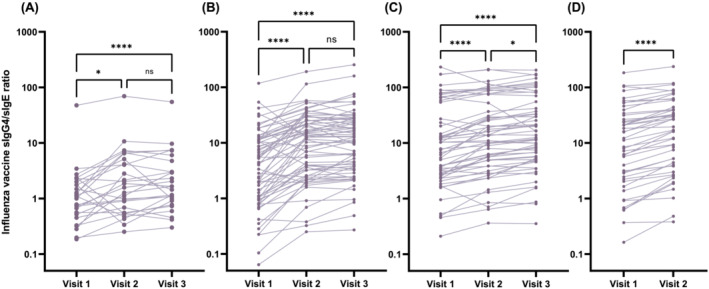
Changes in influenza vaccine‐specific IgG4/IgE ratio before (Visit 1), 4 weeks after the first dose (Visit 2) and the second dose (Visit 3) in the age groups: (A) 6 months to 2 years; (B) 3 to 5 years; (C) 6 to 12 years; and (D) 13 to 18 years. The ratio was significantly elevated in all age groups. *p < 0.05 and ****p < 0.0001; Wilcoxon signed‐rank test or Friedman test, followed by Dunn's multiple comparison test

We then compared the levels of sIgG4, which could function as a blocking antibody to allergic reactions, between the sensitized and non‐sensitized subjects. The IIV‐sIgG4 level was significantly higher in the sensitized subjects than in the non‐sensitized subjects, not only in the 6 months–2 years group (Figure 3A), but also in all age groups (Figure 3B–D). Accordingly, the sIgG4/sIgE ratio increased significantly following vaccination in all age groups (Figure 4). Together, the influenza vaccines induced robust sIgG4 responses, especially in sensitized subjects, which resulted in an increased sIgG4/IgE ratio in healthy children and adolescents who had no allergic AEs following the vaccination.

### 3.8 Patients with anaphylaxis had a low IgG4/IgE ratio

**FIGURE 5:**
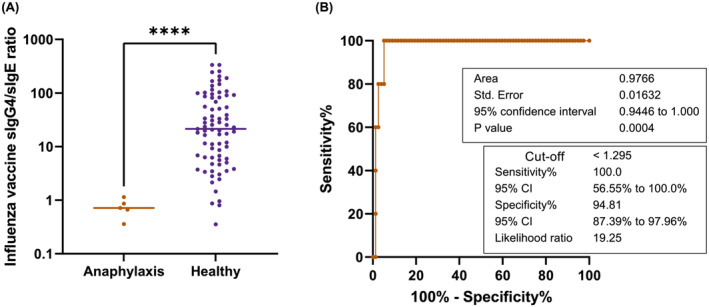
(A) Comparison of influenza vaccine‐specific IgG4/IgE ratio between earlier patients who developed anaphylaxis after influenza vaccination on a separate occasion and age‐matched subjects in the present cohort. Bars indicate the median. ****p < 0.0001; Mann–Whitney test. (B) Receiver operating characteristic (ROC) curve of influenza vaccine‐specific IgG4/IgE ratio for diagnosis of anaphylaxis

Finally, we investigated the sIgG4/sIgE ratio in five patients who developed anaphylaxis following different influenza vaccinations (Table 2) in comparison with age‐matched healthy subjects in the present study. The IIV‐sIgG4/sIgE ratio in the patient group was significantly lower than in the healthy counterparts (Figure 5A). The receiver operating characteristic (ROC) analysis showed the area under the curve to be 0.9766 with high specificity and sensitivity at a cut‐off of 1.295 (Figure 5B).

**TABLE 2:** Characteristics of patients with anaphylaxis following influenza vaccination

Patient no.	Gender	Age (years)	History of influenza infection	History of allergic disease	Induced symptoms
1	M	6	None	None	Urticaria and wheezing
2	M	9	N/A	Urticaria	Urticaria and cough
3	F	7	None	Dog and cat allergy and insect allergy	Urticaria, wheezing, and cough
4	F	5	None	Atopic dermatitis, allergic rhinitis, and allergic conjunctivitis	Urticaria, wheezing, and cough
5	M	5	Yes	Food allergy and atopic dermatitis	Urticaria, wheezing, and cough

Abbreviation: N/A, not available.

## 4 DISCUSSION

In the present study, we demonstrated that sensitization to IIV was common in a healthy pediatric population and that the IIV‐sIgE level increased significantly following vaccination in young children, namely, in the 6 months–2 years and 3–5 years groups. Contrary to the notion that sensitized individuals might have reduced ability to gain protective immunity from vaccines, the sensitized young children had a significantly higher HI titer and IIV‐sIgG level after vaccination. Most importantly, the IIV‐sIgG4 level was significantly higher in sensitized subjects than in non‐sensitized subjects in all age groups, leading to a significant increase in the sIgG4/sIgE ratio following vaccination. In addition, the sIgG4/sIgE ratio was significantly lower in patients with IVA than in age‐matched healthy subjects. These results suggest that IgE sensitization to IIV may be only one aspect of a robust immune response in healthy children, and induction of IgG4 antibodies to IIV may serve as a mechanism to protect them from anaphylaxis.

The presence of IgE antibodies to IIV was first identified in a case‐series study.^7^ Later, we used ELISA to measure the IgE antibody titers in 117 healthy children following 2011/12 TIV.^5^ The influenza antigens used in that ELISA system were A/HIN1/California/07/2009 (2011/12 vaccine strain), A/H1N1/Brisbane/59/2007 (2009/10 vaccine strain), A/H3N2/Uruguay/716/2007 (2009/10 vaccine strain), and B/Brisbane/60/2008 (2011/12 vaccine strain). We demonstrated similar IgE responses to all four strains, regardless of whether the strains were contained in the TIV, with a twofold increase in 33%–56%, 23%–30%, 11%–16%, and 3%–11% of children in 6 months–3 years, 4–6 years, 7–9 years, and 10 years and older groups, respectively, with different percentages depending on the strain. None of those children experienced allergic AEs following vaccination. These results, together with our current study, indicate that IgE sensitization to IIV is not an unusual event in a healthy pediatric population and that IIVs can induce IgE.

In our current study, factors associated with IgE sensitization were age, 3–5 years old, and allergic rhinitis. Proportion of IgE sensitization was highest in the age group and decreased with increasing age. Natural desensitization is observed in some allergens such as food^14^ and mosquito saliva^15^ with unknown mechanisms. Our observation may be analogous to the phenomenon. We assume that allergic rhinitis was detected as a risk factor because it represents atopic predisposition that are susceptible to allergen sensitization. High prevalence of rhinitis in the subject population also contributed to high statistical power.

Because influenza infection often causes asthma exacerbation, the Advisory Committee on Immunization Practices, United States, recommends that all adults and children aged ≥6 months with asthma receive an influenza vaccination annually,^16^ even during the COVID‐19 pandemic.^17^ An important concern had been whether influenza vaccines would provide sufficient immunity in asthma patients, but it was reported that asthma patients' antibody production was sufficient.^18^ However, there have been no reports of comparison of the protective antibody responses between asthma/allergic subjects and their healthy counterparts. In this study, contrary to grim expectations,^13^ we found that HI titers following vaccination were significantly higher in vaccine‐sensitized young children than in non‐sensitized subjects. These results suggest that production of IgE antibodies to influenza vaccine may be a normal, robust, immune response.

Yet binding of IgE to a vaccine component can cause mast cell/basophil activation and pose a risk of developing anaphylaxis after vaccination.^4^, ^8^ Why did none of our present subjects develop any allergic reactions, despite a high rate of sensitization to the vaccine? Perhaps it was because the sensitized subjects had higher IIV‐sIgG4 levels than the non‐sensitized subjects. IgG4 behaves as a monovalent antibody because of dynamic Fab arm exchange^19^; it thus lacks the ability to cross‐link target antigens, has high antigen‐binding affinity.^20^ IgG4's low affinity for Fc receptors^21^ limits its ability to activate inflammatory cells, including mast cells. Collectively, IgG4 antibodies function as “blocking” antibodies against allergic reactions.^22^, ^23^

The concurrent elevation of sIgG4 and sIgE seen in this study was expected because immunoglobulin class‐switching to IgG4 and IgE is dependent on Th2 cytokines such as IL‐4 and IL‐13.^24^, ^25^, ^26^, ^27^ Yet our current results showed that production of sIgG4 exceeded sIgE in our healthy pediatric population, which corresponded to a significant increase in the sIgG4/IgE ratio after vaccination. On the contrary, the patients who developed anaphylaxis following the influenza vaccination had a significantly lower sIgG4/sIgE ratio compared with the healthy subjects. These facts may be attributed to IL‐10 and IL‐10 producing regulatory T cells^28^, ^29^ and regulatory B cells^30^ that promote IgG4 production and inhibit IgE production. In fact, we examined cytokine production in whole blood cultures for some of the subjects in this study and observed significant post‐vaccination production of IL‐10 and IL‐4.^31^ Importantly, IL‐10 production was observed not only in naïve subjects but also in primed subjects (possessing neutralizing antibodies prior to vaccination), whereas IL‐4 production was seen only in naïve subjects,^31^ indicating that IL‐10 is induced in healthy children regardless of their immunological priming status. On the other hand, the anaphylaxis patients may have had impaired IL‐10 production or regulatory immune functions, resulting in impaired production of IgG4 that would mitigate IgE‐mediated allergic reactions.

This study has several limitations. First, we focused only on children and adolescents, not on adults and the elderly. Inclusion of adults and elderly persons might have affected the overall study outcome. In fact, adults were reported to lack IgG4 induction after vaccination.^32^ Therefore, future studies evaluating IgE sensitization and immune responses to influenza vaccines should include adults and the elderly. Second, the study was not designed to identify risk factors for vaccine‐induced anaphylaxis. Although we implied possible involvement of impaired regulatory immune responses in anaphylaxis patients, we did not measure IL‐10 or regulatory T cells in association with IgG4. Further studies are needed to identify pathological immune responses that cause anaphylaxis. Third, we were unable to determine if a propensity for IgE (and IgG4) induction is unique to IIV. To date, only two studies measured IgE (and IgG4) production in response to diphtheria and tetanus toxoids in a healthy population^33^, ^34^; no studies have tested for other vaccines. Although IgG4 may protect from anaphylaxis because of IgE production, vaccines that do not induce IgE may be safer and ideal.

In conclusion, our study demonstrated that IIVs induced not only IgE sensitization but also robust IgG4 responses in healthy children, and that IgE‐sensitized young children had higher HI titers than non‐sensitized children. These results indicate that IgE production with concomitant IgG4 production may represent normal immune responses to influenza vaccination. Because the sIgG4/sIgE ratio was very low in patients who developed anaphylaxis, it may serve as a biomarker for vaccine‐induced anaphylaxis.

## CONFLICTS OF INTEREST

The authors declare the following financial interests/personal relationships that may be considered as potential competing interests. Shigeru Suga reports that financial support was provided by Chemo‐Sero‐Therapeutic Research Institute, Daiichi Sankyo Co., Ltd., the Research Foundation for Microbial Diseases of Osaka University, and Denka Co., Ltd. Kiyosu Taniguchi reports a relationship with Sanofi that includes consulting or advisory. Takao Fujisawa reports having relationships with GlaxoSmithKline, Maruho, MSD, Torii Pharmaceutical, AbbVie, Novartis Pharma, Sanofi, BML Inc., and Pfizer that include consulting or advising, funding grants, and speaking and lecture fees. The rest of the authors declare that they have no relevant conflicts of interest.

## AUTHOR CONTRIBUTIONS

**Prince Baffour Tonto:** Formal analysis. **Mizuho Nagao:** Conceptualization; data curation; formal analysis; investigation. **Shigeru Suga:** Conceptualization; funding acquisition; investigation; project administration. **Kiyosu Taniguchi:** Supervision. **Masahiro Hirayama:** Supervision. **Tetsuo Nakayama:** Conceptualization; investigation; supervision. **Takuji Kumagai:** Conceptualization; investigation; supervision. **Takao Fujisawa:** Conceptualization; formal analysis.

### PEER REVIEW

The peer review history for this article is available at https://publons.com/publon/10.1111/irv.13053.

## Supporting information


**Table S1.** Systemic and local reactions experienced within 7 days after vaccination with TIV and QIV
**Table S2.** Systemic and local reactions experienced in 3 age groups within 7 days after vaccination
**Table S3.** Hemagglutination inhibition (HI) geometric mean titer (GMT) at 28 days post‐vaccination
**Table S4.** Hemagglutination inhibition (HI) geometric mean ratio (GMR) at 28 days post‐vaccination
**Table S5.** Seroconversion rate (SCR) at 28 days post‐vaccination
**Table S6.** Seroprotection rate (SPR)^#^ at 28 days post‐vaccination
**Table S7.** Logistic regression exploring the association between IgE sensitization and age group, gender, history of influenza infection, previous influenza vaccination and physician‐diagnosed allergic diseases

**Figure S1** Specific IgE antibodies to TIV and QIV. Comparison between TIV‐ and QIV‐specific IgE (sIgE) levels before (Visit 1) and at 4 weeks after the 1st dose (Visit 2) and 2nd dose (Visit 3) in each age group: **A,** 6 months to 2 years; **B,** 3 to 5 years; **C,** 6 to 12 years; and **D,** 13 to 18 years. There was no significant difference between the TIV‐ and QIV‐sIgE levels in any of the age groups. Bars indicate geometric mean with 95% confidence interval. Gridlines indicate the lower limit of detection of sIgE. Differences in sIgE levels between TIV and QIV were evaluated by the Mann–Whitney test.

**Figure S2** Antibody responses to the influenza vaccines. Hemagglutination inhibition (HI) antibody titers (measured by HI assay) to H3N2, B Yamagata and Victoria at 4 weeks after the 2nd vaccine dose in each age group: **A, E, I,** 6 months to 2 years; **B, F, J,** 3 to 5 years; **C, G, K,** 6 to 12 years; and **D, H, L,** 13 to 18 years. The levels of HI antibodies to H3N2 and B Yamagata increased significantly in sensitized subjects compared with non‐sensitized subjects in the 6 m‐2y group. Gridlines indicate putative protection level against influenza infection. Mann–Whitney test, ****P < .0001.

## Data Availability

The data supporting the findings of this study are available upon request from the corresponding author.
