# Supplementary material for: High prevalence of IgE sensitization to inactivated influenza vaccines, yet robust IgG4 responses, in a healthy pediatric population
Source: Influenza Other Respir Viruses. 2022 Sep 9;17(1):e13053. doi: 10.1111/irv.13053 (PMC9835421; doi:10.1111/irv.13053)
Supplement: Supplementary file 1 — Table S1. Systemic and local reactions experienced within 7 days after vaccination with TIV and QIV Table S2. Systemic and local reactions experienced in 3 age groups within 7 days after vaccination Table S3. Hemagglutination inhibition (HI) geometric mean titer (GMT) at 28 days post‐vaccination Table S4. Hemagglutination inhibition (HI) geometric mean ratio (GMR) at 28 days post‐vaccination Table S5. Seroconversion rate (SCR) at 28 days post‐vaccination Table S6. Seroprotection rate (SPR)# at 28 days post‐vaccination Table S7. Logistic regression exploring the association between IgE sensitization and age group, gender, history of influenza infection, previous influenza vaccination and physician‐diagnosed allergic diseases [file IRV-17-e13053-s003.docx]

**TABLE E1.** Systemic and local reactions experienced within 7 days after vaccination with TIV and QIV

|  | TIV  n=148 | QIV  n=245 | *P* value^#^ |
| --- | --- | --- | --- |
| Systemic reactions, n (%) | | | |
| Fever | 8 (5.4%) | 17 (6.9%) | .6713 |
| Headache | 10 (6.8%) | 15 (6.1%) | .8329 |
| Fatigue | 11 (7.4%) | 20 (8.2%) | .8492 |
| Anaphylaxis | 0 | 0 | 0 |
| Local reactions, n (%) | | | |
| Itching | 21 (14.2%) | 41 (16.7%) | .5689 |
| Swelling | 40 (27.0%) | 69 (28.2%) | .9075 |
| Injection site pain | 59 (39.9%) | 100 (40.8%) | .9156 |
| Redness | 50 (33.8%) | 82 (33.5%) | .9999 |
| Burning sensation | 28 (18.9%) | 55 (22.5%) | .4456 |

# Chi-square test.

**TABLE E2.** Systemic and local reactions experienced in 3 age groups within 7 days after vaccination

|  | 0-2y  n=96 | 3-5y  n=100 | 6-12y  n=100 | 13-18y  n=97 | *P* value^#^ |
| --- | --- | --- | --- | --- | --- |
| Systemic reactions, n (%) | | | | | |
| Fever | 12 (12.5%) | 6 (6.0%) | 4 (4.0%) | 3 (3.1%) | .0325 |
| Headache | 0 | 2 (2.0%) | 14 (14.0%) | 9 (9.3%) | .0001 |
| Fatigue | 3 (3.1%) | 3 (3.0%) | 15 (15.0%) | 10 (10.3%) | .0029 |
| Local reactions, n (%) | | | | | |
| Itching | 11 (11.5%) | 20 (20.0%) | 23 (23.0%) | 8 (8.3%) | .0131 |
| Swelling | 19 (19.8%) | 28 (28.0%) | 41 (41.0%) | 21 (21.7%) | .0035 |
| Injection site pain | 13 (13.5%) | 41 (41.0%) | 65 (65.0%) | 40 (41.2%) | < .0001 |
| Redness | 22 (22.9%) | 39 (39.0%) | 43 (43.0%) | 28 (28.9%) | .0109 |
| Burning sensation | 14 (14.6%) | 26 (26.0%) | 28 (28.0%) | 15 (15.5%) | .0352 |

# Chi-square test.

**TABLE E3.** Hemagglutination inhibition (HI) geometric mean titer (GMT) at 28 days post-vaccination

| Age group | Vaccine | A/H1N1 | A/H3N2 | B/Yamagata | B/Victoria |
| --- | --- | --- | --- | --- | --- |
| 6m–2y | TIV | 64.3 | 76.9 | 12.2 | 6.0 |
|  | QIV | 47.0 | 72.9 | 13.7 | 13.8 |
| 3–5y | TIV | 110 | 326.1 | 16.0 | 10.6 |
|  | QIV | 120.2 | 302.9 | 25.2 | 26.9 |
| 6-12y | TIV | 157.3 | 309.1 | 28.8 | 20.7 |
|  | QIV | 142.8 | 358.5 | 32.6 | 44.3 |
| 13-18y | TIV | 156.9 | 339.0 | 42.4 | 29.4 |
|  | QIV | 198.6 | 272.9 | 44.8 | 38.2 |

**TABLE E4.** Hemagglutination inhibition (HI) geometric mean ratio (GMR) at 28 days post-vaccination

| Age group | Vaccine | A/H1N1 | A/H3N2 | B/Yamagata | B/Victoria |
| --- | --- | --- | --- | --- | --- |
| 6m–2y | TIV | 7.5 | 9.4 | 2.1 | 1.1 |
|  | QIV | 7.1 | 8.2 | 2.5 | 2.7 |
| 3–5y | TIV | 3.1 | 5.8 | 2.5 | 1.6 |
|  | QIV | 4.2 | 4.8 | 3.5 | 2.9 |
| 6-12y | TIV | 2.8 | 2.8 | 1.9 | 1.5 |
|  | QIV | 2.6 | 3.2 | 2.0 | 2.9 |
| 13-18y | TIV | 2.2 | 2.7 | 1.6 | 1.3 |
|  | QIV | 1.87 | 2.4 | 1.6 | 1.9 |

**TABLE E5**. Seroconversion rate (SCR) at 28 days post-vaccination

| Age group | Vaccine | A/H1N1 | A/H3N2 | B/Yamagata | B/Victoria |
| --- | --- | --- | --- | --- | --- |
| 6m–2y | TIV | 62.9 | 71.4 | 14.3 | 0.0 |
|  | QIV | 63.3 | 71.7 | 13.3 | 18.3 |
| 3–5y | TIV | 56.8 | 67.6 | 21.6 | 5.4 |
|  | QIV | 65.1 | 69.8 | 33.3 | 31.7 |
| 6-12y | TIV | 35.0 | 50.0 | 15.0 | 10.0 |
|  | QIV | 37.7 | 50.8 | 19.7 | 36.1 |
| 13-18y | TIV | 25.0 | 41.7 | 16.7 | 8.3 |
|  | QIV | 21.3 | 37.7 | 14.8 | 16.4 |

**TABLE E6.** Seroprotection rate (SPR)^#^ at 28 days post-vaccination

| Age group | Vaccine | A/H1N1 | A/H3N2 | B/Yamagata | B/Victoria |
| --- | --- | --- | --- | --- | --- |
| 6m–2y | TIV | 65.7 | 74.3 | 17.1 | 5.7 |
|  | QIV | 65.0 | 76.7 | 16.7 | 18.3 |
| 3–5y | TIV | 86.5 | 97.3 | 24.3 | 10.8 |
|  | QIV | 93.7 | 95.2 | 38.1 | 46.0 |
| 6-12y | TIV | 95.0 | 95.0 | 47.5 | 32.5 |
|  | QIV | 91.8 | 100.0 | 52.5 | 70.5 |
| 13-18y | TIV | 94.4 | 97.2 | 72.2 | 50.0 |
|  | QIV | 95.1 | 98.4 | 68.9 | 65.6 |

# Seroprotection level of HI titer ≥40

**TABLE E7.** Logistic regression exploring the association between IgE sensitization and age group, gender, history of influenza infection, previous influenza vaccination and physician-diagnosed allergic diseases

|  | Univariate analysis | | Multivariate analysis | |
| --- | --- | --- | --- | --- |
| Variable | OR (95% CI) | *P* value | OR (95% CI) | *P* value |
| Age groups | | | | |
| *6m-2y* | Reference | | | |
| *3-5y* | 3.577(1.965-6.514) | .000 | 3.584 (1.701-7.549) | .001 |
| *6-12y* | 2.098 (1.186-3.711) | .011 |  |  |
| *13-18y* | 1.314 (0.745-2.318) | .345 |  |  |
| Male sex | 1.001 (0.671-1.494) | .994 |  |  |
| History of influenza infection | | | | |
| *No infection* | Reference | |  |  |
| *Previous season* | 1.225 (0.653-2.299 | .527 |  |  |
| *Other seasons* | 1.005 (0.606-1.666) | .984 |  |  |
| Previous influenza vaccination | 1.023 (0.994-1.053) | .119 | 1.051 (0.999-1.104) | .053 |
| Physician-diagnosed allergic diseases | | |  |  |
| *Bronchial asthma* | 2.079 (1.302-3.321) | .002 | 1.746 (0.994-3.066) | .053 |
| *Atopic dermatitis* | 1.298 (0.702-2.397) | .405 |  |  |
| *Allergic rhinitis* | 2.084 (1.361-3.194) | .001 | 1.749 (1.020-2.999) | .042 |
| *Food allergy* | 1.437 (0.720-2.868) | 0.304 |  |  |

**TABLE E8.** List of investigators

| Name | Institution |
| --- | --- |
| National Hospital Organization Mie National Hospital, Japan | |
| Shigeru Suga, MD, PhD  (Principal Investigator) | Infectious Disease Center and Department of Pediatrics, National Hospital Organization Mie National Hospital |
| Kiyosu Taniguchi, MD, PhD | Infectious Disease Center and Department of Pediatrics, National Hospital Organization Mie National Hospital |
| Takao Fujisawa, MD, PhD  (Corresponding author and Vaccine Safety Study Group) | Allergy Center and Department of Pediatrics, National Hospital Organization Mie National Hospital |
| Mizuho Nagao, MD, PhD  (Vaccine Safety Study Group) | Allergy Center and Department of Pediatrics, National Hospital Organization Mie National Hospital |
| Prince Baffour Tonto, MMSc  (Vaccine Safety Study Group) | Allergy Center, National Hospital Organization Mie National Hospital, and Mie University Graduate School of Medicine |
| Kitasato University | |
| Tetsuo Nakayama, MD, PhD  (Steering Committee) | Omura Satoshi Memorial Institute, Kitasato University Graduate School of Infection Control Sciences |
| Pediatric Clinics in Japan | |
| Takuji Kumagai, MD, PhD  (Steering Committee) | Kumagai Pediatric Clinic |
| Ritsue Nii, M.D., Ph.D. | Department of Pediatrics, Shiroko Clinic |
| Masahiro Watanabe, M.D., Ph.D. | Suzuka Children’s Clinic |
| Tadashi Matsuda, M.D., Ph.D. | Matsuda Pediatric Clinic |
| Masakazu Umemoto, MD. PhD | Umemoto Children’s Clinic |
| Takashi Kato, M.D., Ph.D. | Kato Pediatric Clinic |
| Kaoru Oguchi, M.D., Ph.D. | Saiwai Children’s Clinic |
| Takao Okafuji, M.D., Ph.D. | Okafuji Pediatric Clinic |
| Eitaro Suzuki, M.D., PhD | Suzuki Pediatric Clinic |
| Mie University Graduate School of Medicine | |
| Masahiro Hirayama, MD, PhD. | Department of Pediatrics, Mie University Graduate School of Medicine |

**FIGURE E1** Specific IgE antibodies to TIV and QIV. Comparison between TIV- and QIV-specific IgE (sIgE) levels before (Visit 1) and at 4 weeks after the 1st dose (Visit 2) and 2nd dose (Visit 3) in each age group: **A,** 6 months to 2 years; **B,** 3 to 5 years; **C,** 6 to 12 years; and **D,** 13 to 18 years. There was no significant difference between the TIV- and QIV-sIgE levels in any of the age groups. Bars indicate geometric mean with 95% confidence interval. Gridlines indicate the lower limit of detection of sIgE. Differences in sIgE levels between TIV and QIV were evaluated by the Mann-Whitney test.

**FIGURE E2** Antibody responses to the influenza vaccines. Hemagglutination inhibition (HI) antibody titers (measured by HI assay) to H3N2, B Yamagata and Victoria at 4 weeks after the 2^nd^ vaccine dose in each age group: **A, E, I,** 6 months to 2 years; **B, F, J,** 3 to 5 years; **C, G, K,** 6 to 12 years; and **D, H, L,** 13 to 18 years. The levels of HI antibodies to H3N2 and B Yamagata increased significantly in sensitized subjects compared with non-sensitized subjects in the 6m-2y group. Gridlines indicate putative protection level against influenza infection. Mann-Whitney test, *****P* < .0001.
