# Supplementary figures and images for: High prevalence of IgE sensitization to inactivated influenza vaccines, yet robust IgG4 responses, in a healthy pediatric population
Source: Influenza Other Respir Viruses. 2022 Sep 9;17(1):e13053. doi: 10.1111/irv.13053 (PMC9835421; doi:10.1111/irv.13053)

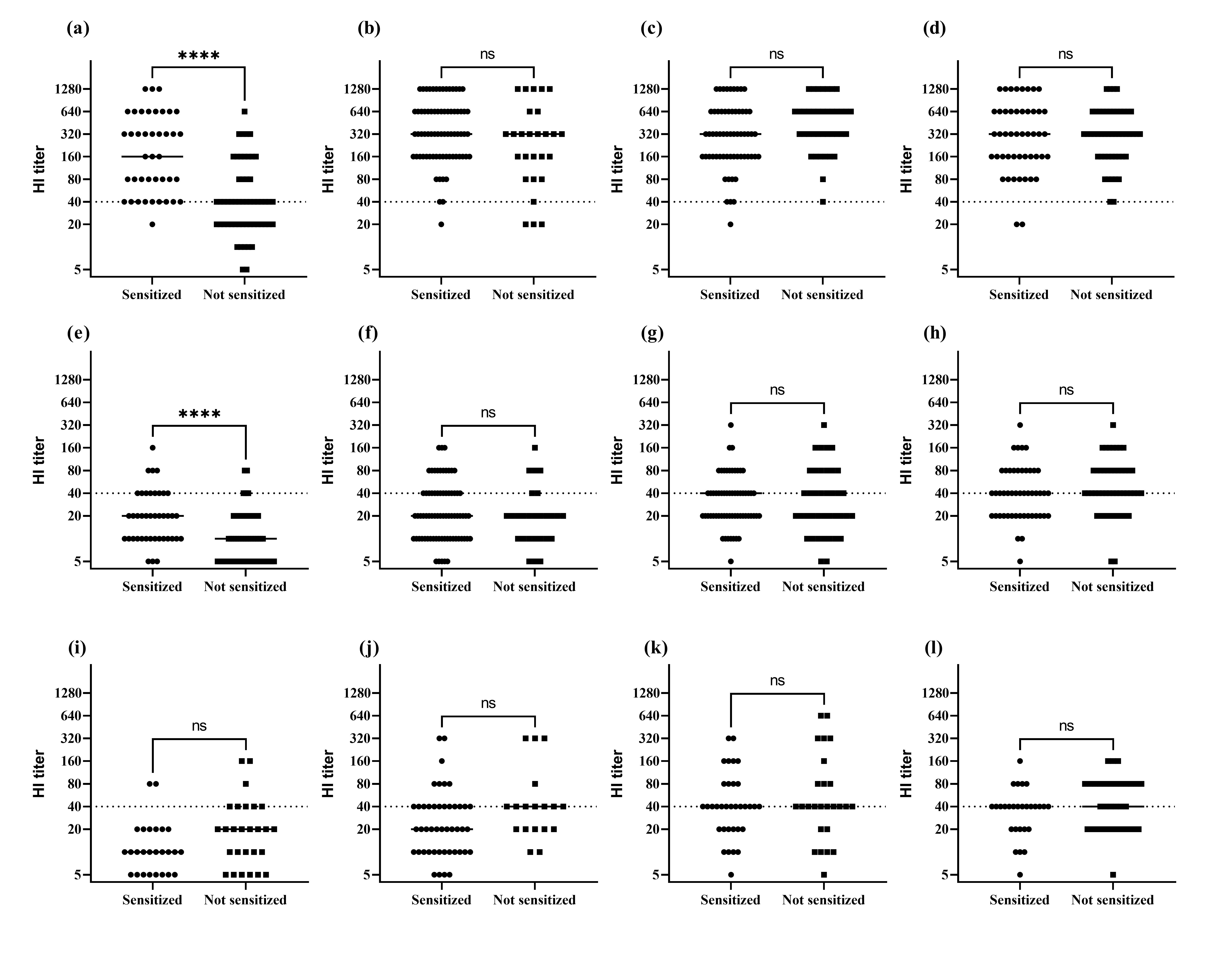

Supplement: Supplementary file 3 — Figure S2 Antibody responses to the influenza vaccines. Hemagglutination inhibition (HI) antibody titers (measured by HI assay) to H3N2, B Yamagata and Victoria at 4 weeks after the 2nd vaccine dose in each age group: A, E, I, 6 months to 2 years; B, F, J, 3 to 5 years; C, G, K, 6 to 12 years; and D, H, L, 13 to 18 years. The levels of HI antibodies to H3N2 and B Yamagata increased significantly in sensitized subjects compared with non‐sensitized subjects in the 6 m‐2y group. Gridlines indicate putative protection level against influenza infection. Mann–Whitney test, ****P < .0001. [file IRV-17-e13053-s002.jpg]
